# Partly Separated Activations in the Spatial Distribution between *de-qi* and Sharp Pain during Acupuncture Stimulation: An fMRI-Based Study

**DOI:** 10.1155/2012/934085

**Published:** 2012-12-24

**Authors:** Jinbo Sun, Yuanqiang Zhu, Lingmin Jin, Yang Yang, Karen M. von Deneen, Wei Qin, Qiyong Gong, Jie Tian

**Affiliations:** ^1^Life Sciences Research Center, School of Life Sciences and Technology, Xidian University, Xi'an, Shaanxi 710071, China; ^2^Huaxi MR Research Center (HMRRC), Department of Radiology, Center for Medical Imaging, West China Hospital of Sichuan University, Chengdu, Sichuan 610041, China; ^3^Institute of Automation, Chinese Academy of Sciences, Beijing 100190, China

## Abstract

Nowadays, functional magnetic resonance imaging (fMRI) has become one of the most important ways to explore the central mechanism of acupuncture. Among these studies, activations around the somatosensory-related brain network had the most robust blood oxygen level-dependent (BOLD) responses. However, due to the insufficient control of the subjective sensations during acupuncture stimulation, whether these robust activations reflected the pattern of *de-qi*, sharp pain, or * mixed * (*de-qi* + sharp pain) sensations was largely unknown. The current study recruited 50 subjects and grouped them into two groups according to whether he/she experienced sharp pain during acupuncture stimulation to give a definite answer to the aforesaid question. Our results indicated that BOLD responses associated with *de-qi* during acupuncture stimulation at ST36 were activation dominated. Furthermore, both the quantitative and qualitative differences of BOLD responses between *de-qi* and mixed sensations evoked by acupuncture stimulation were significant. The pattern of BOLD responses of sharp pain might be partly separated from that of *de-qi* in the spatial distribution. Therefore, we proposed that in order to explore the specific central mechanism of acupuncture, subjects with sharp pain should be excluded from those with only *de-qi*.

## 1. Introduction 

With the aid of functional magnetic resonance imaging (fMRI) techniques, over one hundred studies have been published to explore the neurobiological mechanisms of acupuncture in the past few decades [1–8]. Among these studies, activations around the somatosensory-related brain network had the most robust blood oxygen level-dependent (BOLD) responses in acupuncture [9–11]. Although the majority of early researchers considered that the specific central regulation of acupuncture may be engaged in these activations, recent studies suggested that these results may reflect only an ordinary brain process of the somatosensory stimulation [9] and that acupuncture may turn out to be a kind of deep pain [12]. Therefore, sophisticated studies which form the perspective that acupuncture is a stimulation of the body with multidimensional sensations, focused on exploring the central responses associated with these multi-dimensional sensations evoked by acupuncture stimulation, were urgently needed.

During acupuncture stimulation, multidimensional and intense needling sensations, such as soreness, numbness, distention, heaviness, dull pain, and sharp pain, are experienced by subjects. In general, studies focused on qualification and quantification of needling sensations reached a consensus that *de-qi*, which is traditionally believed to be very important for the possible therapeutic effects of acupuncture, and sharp pain, which is considered to be irrelevant to the acupuncture effect and what acupuncturists try to avoid during needle manipulation, should be quantified separately [13, 14]. However, most previous acupuncture studies in fMRI did not quantify and explicitly distinguish subjects into *de-qi* and sharp pain based on needle sensations, which made striking discrepancies between results of different studies.

Only several studies excluded subjects who experienced sharp pain for fMRI data analysis based on a quantitative needling sensation questionnaire [15–17]. Fewer studies divided subjects into two groups according to whether subjects experienced sharp pain during acupuncture manipulation and compared the BOLD responses between the two groups [6, 18–20]. To make matters worse, significant incompatibilities were shown across the results of these studies. On the one hand, the pattern of BOLD responses of *de-qi* (without sharp pain) evoked by acupuncture stimulation was declared to be activationdominant by some studies but deactivationdominant by others. On the other hand, the pattern of the BOLD responses of mixed sensations (*de-qi* with sharp pain) evoked by acupuncture stimulation was considered to be definite activation by some studies, although these may be two possibilities according to the proportion of different sensations by others. In brief, two core issues of the fMRI-based acupuncture studies are still far from clear. First, what are the *de-qi* related BOLD responses, that is, are they dominated by activation or deactivation? Second, what is the relationship between the *de-qi* related and the sharp pain related BOLD responses?

The current study aims to give a definite answer to these questions. Subjects were grouped into the *de-qi *group or the mixedgroup (*de-qi* + sharp pain) according to whether he/she experienced sharp pain during acupuncture stimulation. The level of the *de-qi* score was compared between groups. The group results of each group and different results/regions between groups were presented. Particularly, the different regions were defined as regions of interest (ROIs) and correlated with the scores from the needling sensations.

## 2. Materials and Methods

### 2.1. Subjects

Participants were recruited from a group of 50 college students (25 males and 25 females; ages 23.3 ± 2.1 years). All subjects were right handed with normal or corrected-to-normal vision. Subjects were acupuncture naïve, had no history of major medical illnesses, head trauma, neuropsychiatric disorders, or any prescription medications one month preceding the experiment, and did not have any contraindications to exposure to a high magnetic field. All subjects gave written and informed consent after the experimental procedures were fully explained. All research procedures were approved by the West China Hospital Subcommittee on Human Studies and were conducted in accordance with the Declaration of Helsinki.

### 2.2. Experimental Procedures

**Figure 1:**
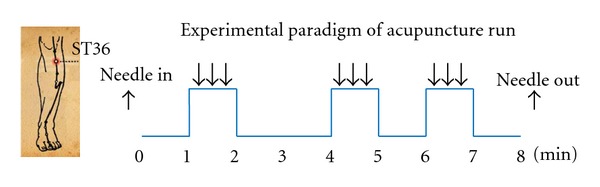
Experimental paradigm. It lasted 8 min and consisted of three one-minute acupuncture stimulations.

For each subject, a functional scanning of the acupuncture stimulation was done. During the scanning, all subjects were instructed to keep their eyes closed to prevent them from actually observing the procedures. Acupuncture stimulation was performed at acupoint ST36 on the right leg (Zusanli, located in the tibialis anterior muscle four fingerbreadths below the lower margin of the patella and one fingerbreadth lateral from the anterior crest of the tibia). The fMRI paradigm for the acupuncture stimulation run lasted 8 minutes and consisted of three one-minute acupuncture manipulations (Figure 1). The needle was inserted perpendicularly to a depth of 2-3 cm before the scan started. A one-minute baseline period was held preceding the first acupuncture stimulation. The interval between the first two acupuncture manipulations was two minutes, while the second and third acupuncture manipulations were separated by an interval of one minute. Scanning was then continued for another minute after the third manipulation. During the acupuncture procedure, the needle was rotated manually clockwise and counterclockwise for one minute at a rate of 60 times per minute. The stimulation was administered by a balanced “tonifying and reducing” technique using a sterile disposable 38 gauge stainless steel acupuncture needle (0.3 mm × 40 mm). After the scan ended, the needle was extracted. In the end, the subjects were facilitated by the acupuncturist to quantify their sensations using a 10-point visual analogue scale (VAS) to rate their *de-qi* experience felt during the acupuncture run, including soreness, numbness, fullness/distention, heaviness, spread, dull pain, and sharp pain. The VAS was scaled as follows: 0, no sensation; 1–3, mild; 4–6, moderate; 7-8, strong; 9, severe; 10, unbearable sensation. Based on the score of sharp pain, 22 subjects were assigned to the *de-qi* group (the score of sharp pain equals zero). The other 28 subjects, with the score of sharp pain being above zero, were assigned to the *mixed* group.

### 2.3. fMRI Scanning Procedure

Imaging data were collected from a 3T Siemens scanner (Allegra, Siemens Medical System) at the Huaxi MR Research Center, West China Hospital of Sichuan University, Chengdu, China. A standard birdcage head coil was used, along with restraining foam pads to minimize head motion and to diminish scanner noise. Thirty axial slices (FOV = 240 mm × 240 mm, matrix = 64 × 64, thickness = 5 mm) parallel to the AC-PC plane covering the whole brain were obtained using a T2*-weighted single-shot, gradient-recalled echo planar imaging (EPI) sequence (TR = 2,000 ms, TE = 30 ms, flip angle = 90°). The scan covered the entire brain including the cerebellum and brainstem. After the functional run, high-resolution structural information on each subject was acquired using 3D MRI sequences with a voxel size of 1 mm^3^ for anatomical localization (TR = 2.7 s, TE = 3.39 ms, matrix = 256 × 256, FOV = 256 mm × 256 mm, flip angle = 7°, in-plane resolution = 1 mm × 1 mm, slice thickness = 1 mm).

### 2.4. fMRI Data Analysis

Preprocessing and statistical analyses at both the individual level and group level were performed using the Statistical Parametric Mapping software (SPM5, http://www.fil.ion.ucl.ac.uk/spm). Initially, the first 5 time points were discarded in order to avoid the instability of the initial MRI signal. The remaining images were realigned to the first volume. Three subjects in the *de-qi* group and five subjects in the *mixed* group exceeded our rigorous motion threshold of less than 1 mm spatial displacement in any direction. Another four subjects in the *mixed* group were deserted randomly in order to balance the number of groups. Ultimately, 19 subjects (8 males) in the *de-qi* group and 19 subjects (9 males) in the *mixed* group remained. Subsequently, the images were normalized to the standard EPI template, resampled to a voxel size of 3 × 3 × 3 mm^3^, and then smoothed spatially using a 6 mm full-width-at-half maximum (FWHM) isotropic Gaussian kernel to decrease spatial noise. Global normalization by proportional scaling was not applied. Then, the time-series from each voxel was high-pass filtered (1/235-Hz cutoff) to remove low-frequency noise and signal drift. For each subject, the preprocessed fMRI data were then submitted for fixed-effects model analyses using the general linear model (GLM) performed at each voxel across the whole brain. After acquiring the contrast images, individual level analyses were accomplished and statistical parametric maps for the *t* statistics (spmT) were then generated for each contrast image. At the group level, the random-effects model analysis was performed based on inference images (i.e., *t* test for contrast images) from the individual level analysis. For exploring the *de-qi* related and *mixed* related BOLD response evoked by acupuncture stimulation, the group results of the one sample *t*-test for each group were mapped and listed at *P* < 0.0001, uncorrected (|*t* | >4.65), and a minimum cluster size of 5 voxels. The different BOLD responses between the *de-qi* and the *mixed* group were explored based on a two sample *t*-test at *P* < 0.0001, uncorrected (|*t* | >4.14), and a minimum cluster size of 5 voxels. Then, the significant regions of the two sample *t*-test were defined as the ROI. In each ROI, the BOLD responses of each subject in the *mixed* group were extracted and correlated with the sharp pain score.

## 3. Results

### 3.1. Psychophysical Results

The average score of sharp pain in the *mixed* group was 1.4 ± 0.7. The sensation of spread was significantly stronger in the *mixed* group (2.2 ± 2.3 versus 4.1 ± 3.0, *P* = 0.03). Other sensations of *de-qi* were comparable between groups (*P* > 0.05; see Table 1 for details). In the *mixed* group, the score of sharp pain and the sum score of *de-qi *were uncorrelated (*r* = 0.25, *P* = 0.30).

### 3.2. Group Results of the **de-qi ** Group

**Figure 2:**
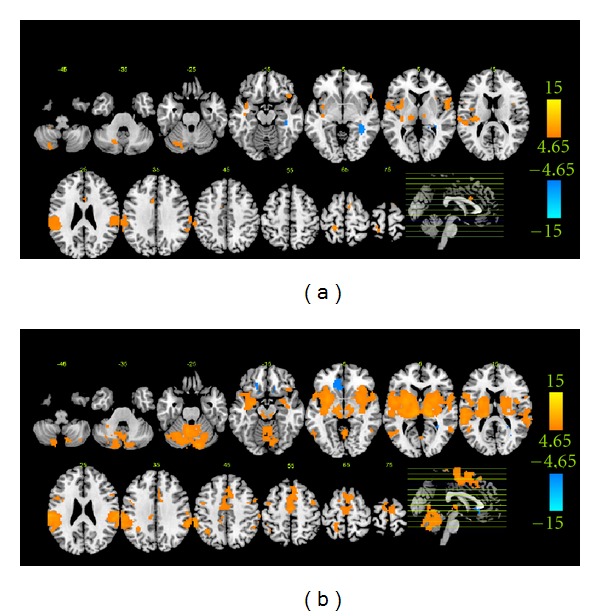
Group-level BOLD responses for each group. Panels (a) and (b) show the groups results of* de-qi* and *mixed* group evoked by acupuncture stimulation at *P* < 0.00001, uncorrected with 5 contiguous voxels, respectively.

Figure 2(a) shows group activations and deactivations of the *de-qi* group evoked by acupuncture stimulation at ST36. The primary somatosensory cortex (SI), the secondary somatosensory cortex (SII), the inferior parietal lobule (IPL), the insula, the thalamus, the cingulate gyrus (Brodmann area (BA) 24/32), the precentral gyrus (BA4/6/44), the inferior frontal gyrus, the putamen, the claustrum, the superior temporal gyrus (STG), and the transverse temporal gyrus and the cerebellum (VI, VIIb, VIII, CrusI, and CrusII) were significantly activated. Most of these activations were bilateral and showed a lateralization to the hemisphere contralateral to the stimulation. Only the ipsilateral parahippocampal gyrus was significantly deactivated. 

### 3.3. Group Results of the Mixed Group

Figure 2(b) shows group activations and deactivations of the *mixed* group evoked by acupuncture stimulation at ST36. Significant activations were presented in the SI, the SII, the IPL, the supramarginal gyrus (SMG), the insula, the thalamus, the putamen, the caudate, the cingulate gyrus (BA24/32), the claustrum, the precentral gyrus (BA4/6/44), the inferior frontal gyrus, the medial frontal gyrus, the middle frontal gyrus, the superior frontal gyrus, the lingual gyrus, the fusiform gyrus, the parahippocampal gyrus, the middle temporal gyrus, the inferior temporal gyrus, the STG, the transverse temporal gyrus, and the cerebellum (see Table 2 for details). Most of these activations were bilateral. The contralateral perigenual anterior cingulate and the contralateral medial frontal gyrus were significantly deactivated. 

### 3.4. Different Results between the **de-qi ** Group and the Mixed Group

**Figure 3:**
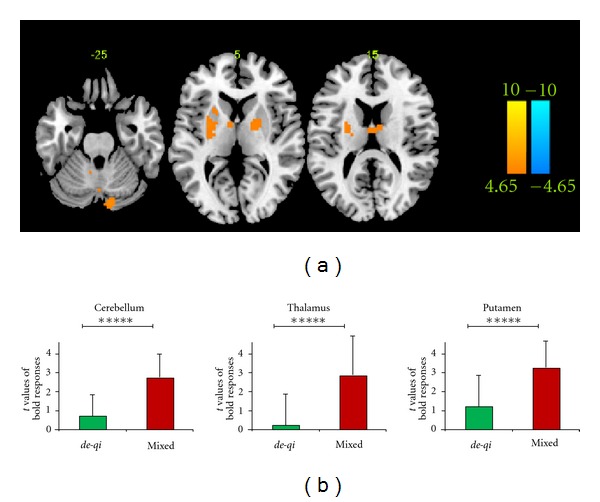
Different BOLD responses between groups. Panel (a) shows the between-group results of “*mixed* > *de-qi*” at *P* < 0.0001, uncorrected with 5 contiguous voxels. Panel (b) shows the mean *t* values of the BOLD responses in each ROI for both groups (*****is equivalent to *P* < 0.00001).

Figure 3 shows the different results between groups. Significantly stronger activations of the *mixed* group were presented in the bilateral putamen, the bilateral thalamus, and the bilateral cerebellum (CrusI and CrusII). No regions showed stronger activations of the *de-qi* group (see details in Table 3). In these regions, BOLD signals of the *de-qi* group were barely changed, while significant BOLD responses were shown in the *mixed* group. The level of the BOLD responses and the intensity of subjective sharp pain was uncorrelated (all *P* > 0.1 uncorrected) in these regions in the *mixed* group (figure not shown).

### 3.5. Different Results between the Weak **de-qi ** Group and the High de-qi Group

For eliminating the possibility that the three discrepant regions in Section 3.4 were due to the different global *de-qi* intensity between groups, we divided the pure *de-qi *group into two subgroups based on the sum score of *de-qi* (weak *de-qi* group: 10 subjects, averaged sum score of *de-qi* was 9.48 ± 3.92; strong *de-qi* group: 9 subjects, averaged sum score of *de-qi* was 20.63 ± 5.35). Although the difference of the *de-qi* intensity between these two subgroups (weak *de-qi* group and strong *de-qi* group) was much greater than that between the two groups (*de-qi* group and *mixed* group), no regions were shown in the intersubgroup results (*P* > 0.01, uncorrected for all voxels, figure not shown). 

## 4. Discussion 

The present study focused on two core issues of the fMRI-based acupuncture studies. Firstly, our results indicated that the *de-qi* related BOLD responses were dominated by activations, mainly around the somatosensory-related brain network. Secondly, our results showed that the *de-qi* + sharp pain evoked by acupuncture stimulation were associated with more extensive and strong activations. Particularly, specific activations of pain-related regions at the part of the bilateral putamen, thalamus, and cerebellum were shown only in the *mixed* group, which presumed that the BOLD response patterns of *de-qi* and sharp pain were partly separated in the spatial distribution. 

### 4.1. Activation-Dominated BOLD Responses Associated with **de-qi ** during Acupuncture Stimulation

Few studies explored the BOLD responses associated with pure *de-qi* (i.e., without sharp pain). In Na et al., 2009, with pure *de-qi*, patterns of BOLD responses to acupuncturing at GB34 were predominately activated [21]. In Bai et al., 2010, with pure *de-qi*, predominantly positive signal changes were seen during acupuncturing at GB37 [22]. In accordance with these previous studies, our results clearly indicated that significant activations mainly in the SI, the SII, the IPL, the insula, the thalamus, the cingulate cortex, and the cerebellum were associated with pure *de-qi* during acupuncture stimulation at ST36 (Figure 2(a) and Table 2). However, our results also conflicted with other previous studies. Hui and colleagues proposed a brain network called the limbic-paralimbic-neocortical network to name the predominant deactivated brain regions associated with pure *de-qi* during acupuncture stimulation [6, 19, 20, 23]. We inferred that several possible reasons might contribute to the deactivation-dominated BOLD response patterns. Firstly, the average of the repeated runs for each subject was applied. Repetition may itself alter the distribution of the activated regions due to the influence of memory and expectation [24]. Secondly, global normalization, a questionable data processing step adopted in several fMRI-based acupuncture studies, can introduce an artificially negative relationship with the task [25–29]. Particularly, our recent study clarified that for the fMRI-based acupuncture's data, global normalization significantly changed the activation-dominated results to deactivation-dominated [29]. Therefore, we suggested that BOLD responses associated with *de-qi* during acupuncture stimulation at ST36 should be activation dominated. 

These *de-qi* related activations were mainly around the somatosensory-related brain network. Previous acupuncture studies tended to believe that these results reflected the specific central regulation of acupuncture [5, 21–23]. However, several recent studies argued that acupuncture stimulation was similar to a special deep pain stimulation [12]. Therefore, the BOLD responses of acupuncture stimulation were considered to mainly reflect the general central processing of a special deep pain stimulation [30]. In contrast to superficial (cutaneous) pain, deep pain (originating from muscle, joints or viscera) is dull, diffuse, and difficult to localize [30, 31]. In Henderson et al., 2006, the researchers used intramuscular injections of hypertonic saline to construct a deep pain model [30]. The subjective sensations under this deep pain mainly included tenderness, heaviness, aching, cramping, throbbing, and gnawing, which were similar to that of *de-qi* evoked by acupuncture stimulation. Activations of deep pain based on intramuscular injections of hypertonic saline were located in the middle cingulate gyrus, insula, SI, SII, motor regions, and cerebellum [24, 30]. Other studies explored the central processing of deep pain based on intramuscular electrical stimulation (IMES) [31] or mechanical pressure on muscle [32]. During IMES, the SI, SII, insula, IPL, precuneus, superior temporal gyrus, cingulate gyrus (anterior, middle, and posterior), claustrum, thalamus, precentral gyrus, and frontal gyrus (inferior, medial, and middle) were activated [31]. Activations of the ACC, insula, SII, IPL, thalamus, putamen, claustrum, and caudate were correlated with the mechanical-pressure-based muscle pain [32]. Most activations of *de-qi* in the present study were reported in the aforementioned deep pain studies, which might indicate similar central processing of the same origin of stimulation and similar subjective sensations. Due to individual variability of brain morphology and differences in experimental design, the central patterns of activation during deep pain and acupuncture stimulation were difficult to compare between studies. Therefore, we suggested that a specific central effect of *de-qi* during acupuncture stimulation might be illustrated after comparing it directly to deep pain stimulation.

### 4.2. Relationship between **de-qi ** Related and Sharp Pain Related Bold Responses

In the domain of pain studies, it is generally known that differences of the quality and origin between sharp pain and deep pain were remarkable [33]. Although one early positron emission tomography (PET) study argued that skin pain and muscle pain had a common representation [34], abundant evidence from animal investigations [35, 36], clinical data [37–39], and human neuroimage studies [24, 30, 31, 40–43] indicated different brain processing of acute superficial pain (sharp pain) and deep pain. For acupuncture, both the deep-pain-like *de-qi* sensations and sharp pain were evoked by identical acupuncture processing. The sharp pain in acupuncture could be due to stimulation of the superficial cutaneous pain fibers or the pain fibers in deep tissues. Therefore, the differences between *de-qi* and sharp pain in acupuncture could possibly be attributed to only the quality of sensation or both the quality and origin of sensation. In either case, certain differences of central responses should be detected. Since subjects with pure sharp pain during acupuncture stimulation were hardly obtained, the difference between *de-qi* and sharp pain evoked by acupuncture stimulation could not be directly presented. Therefore, we had to infer their differences between the *de-qi* and *mixed* groups. When considering all the needling sensations as a whole, activations of the SII, insula, SI, cerebellum, and thalamus were the most robust BOLD responses evoked by acupuncture stimulation in previous acupuncture fMRI studies [9–11]. Consistent results were shown in our study so that extensive activations of cortical areas were relevant to the processing of somatosensory, motor, and pain signals which were associated with *de-qi* + sharp pain evoked by acupuncture stimulation (Figure 2(b) and Table 2). 

Based on the visual comparison of the *de-qi* and *mixed* groups' group results, the activated regions of the *mixed* group mainly covered that of the *de-qi *group and had a larger extent and stronger intensity. Moreover, activations of the frontal gyrus (medial, middle, and superior), temporal gyrus (inferior and middle), SMG, caudate, amygdala, parahippocampal gyrus, lingual gyrus, fusiform gyrus, and numerous subregions in the cerebellum were also activated in the *mixed* group. Most of these visual differences failed to survive in the two-sample *t* test, thus the interpretation of these differences should be taken with caution. We argued that they might reflect the slightly stronger subjective sensations of *de-qi *in the* mixed *group or the common pattern of both* de-qi *and sharp pain, rather than the specific pattern of sharp pain, such as detection of a painful stimulus and/or encoding of pain localization, intensity, and duration [30]. In addition, deactivations of the parahippocampal gyrus and the subgenual cingulate/medial frontal gyrus were unique to the *de-qi* and *mixed* groups, respectively. A previous study showed that the BOLD responses in the hippocampus/parahippocampus were altered depending on the type of tissue stimulated, that is, deactivated during deep pain and activated during superficial pain [24], which accorded with our results and might indicate different emotional and cognitive processing of these two kinds of pain [24, 44, 45]. As part of the default mode network (DMN), the deactivation of the subgenual cingulate/medial frontal gyrus was usually reported in previous acupuncture fMRI studies associated with the mixed group [5, 10, 29], indicating a function to enter a mode of preparedness and alertness for possible changes in the internal or external milieu [5]. However, these two deactivated regions also failed to survive in the two-sample *t* test. The deactivation of the hippocampus or parahippocampus was also found in previous acupuncture fMRI studies associated with the mixed group [5, 29]. Therefore, these differences should also be interpreted with caution.

Based on the two-sample *t *test between groups, parts of the putamen, thalamus, and cerebellum were significantly different (Figure 3 and Table 3). Specifically, in these regions, BOLD signals were barely changed in the *de-qi* group, but they were significantly increased in the *mixed* groups. We suggested that this might reflect the specific brain processing of sharp pain, rather than the different global *de-qi* intensity between groups (see Section 3.5 for details) or the common pattern but with a different degree for the *de-qi* and *mixed* group. The thalamus, as a structure that receives projections from multiple ascending pain pathways, is involved in the sensory discriminative and affective motivational components of pain [46–48]. Particularly, the activations of the thalamus would most be expected in the acute pain state [49], which was consistent with the current study. Since *de-qi* might originate from deep tissues and sharp pain might originate from superficial cutaneous pain fibers, the difference in the thalamus might reflect the different originating structures of pain [30, 31]. As a brain region of the salience network, the putamen was important in the integration of cognitive information [50, 51]. For pain, the putamen might be able to utilize the cognitive context to influence the selection of appropriate brain networks, which were engaged during the inflow of afferent nociceptive information [52]. In the current study, the difference in the putamen might reflect the specific processing of sharp pain. The difference in the cerebellum was located in lobules CrusI and CrusII, which are involved in cognitive processing of pain [53] and might reflect the specific central processing of sharp pain. However, the activities of these discrepant regions were not correlated with the subjective sensations, indicating that they did not encode subjective pain intensity (figure not shown). In short, we suggested that these discrepant regions might be associated with specific perceptual and emotional qualities of sharp pain. 

In particular, another interpretation for these quantitative and qualitative differences that should be discussed is the different salience of *de-qi* and sharp pain. Previous deep pain studies found that the cortices of the temporoparietal junction (BA22/39/40), which included both bilateral STG and SMG, were significantly activated [31]. The temporoparietal junction was considered to respond preferentially to behaviorally relevant stimuli and play a general role in detecting salient stimuli [54–56]. Recently, the “salience network” was dissociated, which enables the integration of highly processed sensory data with visceral, autonomic, and hedonic markers so that the organism can decide what to do (or not to do) next [57–59]. The salience network includes the anterior insula, the anterior cingulate cortex, as well as more limbic and paralimbic structures (such as the amygdala and putamen) and the cerebellum (VI) [52, 57, 59, 60]. Most of these regions were activated in both the *de-qi* and *mixed* groups, which might indicate that both *de-qi* and sharp pain sensations evoked by acupuncture stimulation were salient external stimuli. Furthermore, because sharp pain is often escapable and generally evokes active emotional coping behaviors, while deep pain is usually inescapable and evokes often a passive emotional coping behavior [24, 30], the stronger and more extensive activation in these regions in the *mixed* group, as well as the regions that were activated only in the *mixed* group, might reflect that sharp pain is more salient than deep-pain-like *de-qi* sensations. 

In conclusion, both the quantitative and qualitative differences of BOLD responses between *de-qi* and mixed sensations evoked by acupuncture stimulation were distinct. We inferred that the pattern of BOLD responses of sharp pain might be partly separated from that of *de-qi* in the spatial distribution. The subjects with sharp pain should be excluded from those with only *de-qi*, when exploring the central BOLD responses during acupuncture stimulation.

### 4.3. Limitations

This paper still has space for improvement in upcoming studies. Firstly, the scoring of the subjective sensations during acupuncture stimulation was based on recollection, so the reference vector was strictly based on the “ON-OFF” pattern. Because the sensations elicited by acupuncture stimulation might fail to disappear immediately when the manipulation was stopped, the BOLD responses of the acupuncture stimulation partly remained in the intervals between acupuncture manipulations [4, 61]. Therefore, the interaction effect among manipulations might exist. The real-time scoring in further studies would provide more precise information. Even so, using a relatively conservative threshold, authentic activations (or deactivations) under the “ON-OFF” pattern could be detected. Particularly, the results of this paper mainly reflected the BOLD responses of *de-qi* or *de-qi* mixed with sharp pain evoked by acupuncture stimulation, rather than the effect of acupuncture. Secondly, the control group with pure sharp pain sensation was absent. Since subjects with pure sharp pain were difficult to obtain during acupuncture stimulation, further studies would collect these subjects from a large sample size. Finally, the quantity of the stimulus during the BLOCK-designed manual acupuncture was hardly equivalent. Further studies should quantify the stimulus of acupuncture with assistive devices.

## 5. Conclusions

The current study focused on two core issues of the fMRI-based acupuncture studies. For the first question about what are the *de-qi* related BOLD responses dominated by, our answer was that BOLD responses associated with *de-qi* during acupuncture stimulation at ST36 were activation dominated. These *de-qi* related activations were mainly around the somatosensory-related brain network and were similar to those in previous deep pain studies. Therefore, we suggested that these activations might indicate similar central processing to the same origin of stimulation and similar subjective sensations. For the second question about what is the relationship between *de-qi* related and sharp pain related BOLD responses, our answer was that the pattern of BOLD responses for sharp pain might be partly separated from that of *de-qi* in the spatial distribution. Furthermore, we proposed that subjects with sharp pain should be excluded from those with only *de-qi* when exploring the central BOLD responses during acupuncture stimulation, because both the quantitative and qualitative differences of BOLD responses between *de-qi* and mixed sensations evoked by acupuncture stimulation were significant.

## Figures and Tables

**Table 1:** Scores of needling sensations between groups.

Sensations	VAS scores (mean ± SD)		Significance level
	*de-qi* group (*n* = 19)	Mixed group (*n* = 19)	
Soreness	2.7 ± 2.3	2.7 ± 2.5	*P* > 0.05
Numbness	1.7 ± 2.0	2.9 ± 2.3	*P* > 0.05
Fullness	5.0 ± 1.6	6.3 ± 2.5	*P* > 0.05
Heaviness	2.4 ± 2.9	4.1 ± 2.6	*P* > 0.05
Spread	2.2 ± 2.3	4.1 ± 3.0	**P** = 0.03
Dull pain	0.8 ± 1.4	1.4 ± 1.4	*P* > 0.05
Sharp pain	0	1.4 ± 0.7	

VAS: visual analogue scale; SD: standard deviation.

**Table 2:** Significant activations of each group.

Regions	BA or A/P		*de-qi* group					Mixed group				
			Talairach			*t* value	Voxels	Talairach			*t* value	Voxels
			*x*	*y*	*z*			*x*	*y*	*z*
Cerebrum												
Inferior frontal gyrus	6/9/13/44/45/47	L	−36	8	−11	5.04	8	−33	17	−3	7.18	81
		R	42	20	−14	6.08	16	36	17	−3	6.17	32
Medial frontal gyrus	6/32	L						−3	−9	53	7.40	69
		R						3	−9	50	7.51	68
Middle frontal gyrus	6/9	L						−42	0	50	6.89	18
		R						42	2	50	7.32	33
Superior frontal gyrus	6	L						−3	5	49	6.21	31
		R						3	11	55	8.09	70
Precentral gyrus	6/44	L	−15	−32	65	5.93	18	−48	0	8	9.50	43
		R	50	−2	8	6.16	8	48	0	8	6.56	23
Postcentral gyrus	1/2/3/5/7/40/43	L	−18	−35	65	8.20	65	−62	−22	20	11.04	98
		R	65	−22	31	5.49	17	56	−28	21	9.16	48
Inferior parietal lobule	40	L	−65	−28	32	7.43	62	−65	−25	23	9.52	151
		R	59	−28	26	6.41	38	65	−36	29	9.33	85
Supramarginal gyrus	40	L						−53	−46	22	7.30	24
		R						59	−39	32	9.33	15
Thalamus		L	−12	−20	7	5.90	44	−15	−23	9	10.55	238
		R	9	−17	4	6.06	22	15	−23	7	11.02	189
Insula	13/40/41/47	L	−33	−23	12	7.26	58	−56	−34	18	7.89	153
		R	39	6	11	5.15	6	42	1	11	7.21	98
Claustrum		L	−33	−20	9	7.62	24	−30	9	−3	9.19	55
		R						30	11	−3	8.28	52
Cingulate gyrus	24/32	L	−6	13	32	6.01	14	−3	−1	47	6.99	56
		R	6	22	27	6.02	5	6	20	40	6.54	56
Caudate		L						−15	4	14	5.97	25
		R						36	−15	−7	6.82	26
Putamen		L	−30	−17	9	6.02	6	−27	0	8	9.88	284
		R						24	3	5	8.16	255
Amygdala		L						−27	−4	−12	7.73	17
Lingual gyrus	18	L						−15	−82	−11	5.54	7
		R						18	−79	−14	7.25	11
Fusiform gyrus	19	L						−21	−79	−14	5.41	8
		R						21	−79	−14	6.03	7
Middle temporal gyrus	21/22/37/39	L						−56	−64	3	6.85	20
		R						56	−52	11	5.93	4
Inferior temporal gyrus	19/37	L						−53	−64	1	5.94	7
Superior temporal gyrus	13/22/38/41/42	L	−59	3	3	6.14	38	−59	−34	18	9.13	150
		R	53	6	2	6.10	8	56	0	3	8.01	90
Transverse temporal gyrus	41/42	L	−39	−23	12	5.51	6	−45	−23	12	7.79	22
		R						62	−17	12	5.38	14

Cerebellum												
Cerebellum_4_5	A	L						−6	−53	−15	7.93	31
		R						9	−50	−13	5.95	22
Cerebellum_6	A	L						−27	−56	−20	6.89	60
		R						9	−59	−10	5.47	25
	P	L	−18	−62	−22	5.98	24	−27	−59	−20	9.07	124
		R						24	−62	−20	7.00	113
Cerebellum_7b	P	L	−24	−72	−34	5.55	6	−15	−75	−37	6.83	27
		R						9	−75	−34	8.11	13
Cerebellum_8	A	L						−6	−62	−25	6.48	12
	P	L	−18	−65	−27	6.36	8	−9	−62	−22	7.33	32
		R						9	−72	−37	6.89	14
Cerebellum_Crus1	A	L						−36	−56	−22	5.89	14
		R						36	−51	−25	4.89	5
	P	L	−18	−68	−24	6.37	54	−15	−68	−19	8.02	173
		R						12	−83	−19	9.34	88
Cerebellum_Crus2	P	L	−24	−75	−34	5.65	11	−12	−77	−21	7.96	148
		R						12	−85	−18	10.23	94
Vermis_3	A	R						3	−44	−15	5.21	7
Vermis_4_5	A	L						0	−58	−2	7.27	46
		R						3	−61	−2	6.22	55
Vermis_6	A	L						−3	−56	−15	6.51	18
		R						0	−56	−15	6.24	20
	P	L						−3	−71	−12	5.27	18
		R						3	−59	−17	6.86	25
Vermis_7	P	L						−3	−62	−17	6.96	13
		R						0	−62	−17	6.69	26
Vermis_8	A	L						−3	−62	−25	6.45	10
		R						3	−56	−20	6.72	14
	P	L						0	−65	−27	6.31	19
		R						3	−68	−29	6.76	26
Vermis_9	A	L						0	−57	−25	6.15	6
		R						3	−56	−22	6.61	8
Vermis_10	A	L						−3	−51	−20	6.81	5
		R						0	−51	−20	6.35	8

The coordination of voxel with the maximal *t* within each region is listed. The regions are thresholded at *P* < 0.0001, uncorrected.

BA: Brodmann area; L: left; R: right; A: anterior lobe; P: posterior lobe.

**Table 3:** Significant difference between groups (*mixed* > *de-qi*).

Regions			Talairach			*t* value	Voxels
			*x*	*y*	*z*
Cerebrum							
Thalamus		L	−6	−8	11	5.11	19
		R	6	−5	11	4.93	9
Putamen		L	−27	−2	11	5.10	53
		R	21	0	6	4.78	32

Cerebellum							
Cerebellum_Crus1	A	L					
		R					
	P	L					
		R	12	−83	−19	5.75	20
Cerebellum_Crus2	A	L					
		R					
	P	L	−9	−86	−23	4.95	6
		R	9	−83	−19	6.11	12

The coordination of voxel with the maximal *t* within each region is listed. The regions are thresholded at *P* < 0.0001, uncorrected.

BA: Brodmann area; L: left; R: right; A: anterior lobe; P: posterior lobe.
